# Distribution and Transmission of Medicinal Plant Knowledge in the Andean Highlands: A Case Study from Peru and Bolivia

**DOI:** 10.1155/2012/959285

**Published:** 2011-11-29

**Authors:** Sarah-Lan Mathez-Stiefel, Ina Vandebroek

**Affiliations:** ^1^Centre for Development and Environment, University of Bern, Hallerstrasse 10, 3012 Berne, Switzerland; ^2^Institute of Economic Botany, The New York Botanical Garden, 2900 Southern Boulevard, Bronx, NY 10458, USA

## Abstract

This paper presents a study of patterns in the distribution and transmission of medicinal plant knowledge in rural Andean communities in Peru and Bolivia. Interviews and freelisting exercises were conducted with 18 households at each study site. The amount of medicinal plant knowledge of households was compared in relation to their socioeconomic characteristics. Cluster analysis was applied to identify households that possessed similar knowledge. The different modes of knowledge transmission were also assessed. Our study shows that while the *amount* of plant knowledge is determined by individual motivation and experience, the *type* of knowledge is influenced by the community of residence, age, migratory activity, and market integration. Plant knowledge was equally transmitted vertically and horizontally, which indicates that it is first acquired within the family but then undergoes transformations as a result of subsequent contacts with other knowledge sources, including age peers.

## 1. Introduction

The social processes of acquisition and transmission of knowledge, which are unique to each culture, shape local (environmental) knowledge (hereafter LK) defined as “a cumulative body of knowledge, practices, and beliefs, evolving by adaptive processes and handed down through generations by cultural transmission” [1, page 1252] and [2, page 8]. While the transmission of LK was still considered a rather neglected field at the end of the 1990s [3], this is no longer the case today. An increased number of studies on the processes of transmission and acquisition of LK have been published in recent years, often linked to growing concerns over its loss [4–8]. Zent, for instance, states that the “persistence and resilience [of LK] over time is critically dependent upon (…) customary methods of knowledge transmission” [9, page 104]. Several authors use a model of cultural transmission that was first developed by Cavalli-Sforza and Feldman [10] and later refined by Hewlett and Cavalli-Sforza [11] (see also [4, 5, 8, 12]). This model describes four modes of cultural transmission, understood as “a process of social reproduction in which a culture's technological knowledge, behavior patterns, and cosmological beliefs are communicated and acquired” [11, page 922] together with their implications for cultural evolution: (1) vertical (parent-to-child, characterized as being highly conservative with slow cultural evolution and high intracultural variation, (2) horizontal (between unrelated individuals in which cultural evolution can be rapid and intracultural variation can be high), (3) one-to-many (as between a teacher and pupils, where communication is highly efficient, cultural evolution is most rapid, and intracultural variation is low), and (4) concerted or many-to-one (between the older and the younger members of social groups; this type of transmission is most conservative, and shows very slow cultural evolution and very low intracultural variation) [11]. Cultural transmission usually occurs through various mechanisms, depending on the context, whose relative weight must be evaluated to assess the stability of cultural traits over time and space [8].

Intracultural variation is another key contemporary field of inquiry related to LK and ethnobotanical research [13–27]. Intracultural variation is “patterned according to differences in individual experience and access to knowledge” [22, page 335]. These individual differences are determined by several factors such as age, gender, kinship relations, schooling, occupation, and contacts with other actors and sources of knowledge. Interest in discerning the patterns of intracultural variation is twofold. On the one hand, these patterns allow us to identify relationships with social factors of cultural change [28], thus providing information about processes of transformation of LK. On the other hand, they make it possible to infer how learning takes place [29] (cited by [15]), and hence to understand processes of knowledge transmission and acquisition. Nevertheless, studies on the distribution of LK have often produced unclear and sometimes differing results. For instance, most of the literature stresses the negative relationship between schooling and LK [9, 13, 15, 16, 18, 21, 30]. These authors generally highlight the influence of the role of acculturation through state-run education, which is considered an “intrusive knowledge form” [21] that competes with the acquisition of LK. However, the negative association between schooling and LK is low when school curricula are culturally contextualized [30]. Furthermore, a few cases are reported where some individuals with higher schooling had higher LK competence than their peers [3, 17, 19]. Zent, for example, states that the higher ethnobotanical scores of some children with higher schooling “may also be influenced by individual motivation and/or natural intelligence, since usually only motivated, smart people reach these upper educational levels” [31, page 11] (cited by [19]). Regarding the effect of migration on ethnobotanical knowledge, Pieroni and Vandebroek [32] describe two opposite explanatory concepts: (1) lower LK as a consequence of cultural adaptation and acculturation and (2) equal or higher LK due to resilience and strengthening of cultural identity. According to these authors, adaptation, as a result of cultural negotiations, is only one of the possible strategies migrants might adopt during their interaction with the host culture, whereas an opposite strategy might be one aimed at strengthening their cultural identities. While the first strategy would probably imply a loss of traditional knowledge and use of plants, the latter set of strategies might lead to a deliberate retention of ethnobotanical practices. In their study on knowledge of forest plants among a returnee community in Guatemala, Nesheim et al. [33] showed that migration can lead to changes in consumption patterns, and thus to the replacement of LK. However, they also demonstrated that some domains of LK, such as knowledge about medicinal plants, were maintained. The influence of the market economy on LK is another question that has interested ethnobiologists. Several researchers have stressed the danger that the global economy represents for LK, by leading to deep modifications of local management systems [34, 35]. According to these authors, the market economy threatens the social reciprocal exchanges by transforming nonmonetary values into monetary values. Another explanation is given by Reyes-García et al., who state that “the development of market economies tends to be correlated with greater socioeconomic heterogeneity, and therefore one might expect greater variance in plant knowledge as markets expand.” [36, page 651]. Studies of the correlation between LK and market-related activities have, nevertheless, produced ambiguous or even contradictory results [16, 36].

In the Andes, LK has been described as being highly diverse and place specific, and transmitted from one generation to the next through cultural transmission [7]. In the last two decades, several development cooperation and research projects have been actively engaged in mitigating the loss of cultural diversity and LK in the Andean highlands, through activities such as the revalorization of Andean environmental knowledge and worldviews and implementation of bilingual and intercultural education programs [7, 37–40]. It is believed that the presumed erosion of Andean environmental knowledge is a consequence of the current deep transformations of Andean society resulting from an increasing process of “modernization”, as represented by the state-run education system [7, 41, 42], the market economy [37, 41], migration to urban centers [37, 42], and the presence of primary health care services [41, 42]. Conversely, some researchers have highlighted the resilience of Andean knowledge, presented as being capable of reacting to change and conflict through mechanisms of “creative resistance” [43] or resilient adaptation and transformation [44].

As part of a larger research project on the processes of transformation in Andean medicinal knowledge, this paper deals with the patterns of distribution and transmission of medicinal plant knowledge in rural communities of the Peruvian and Bolivian Andes. Research questions to be answered include (1) what are the socioeconomic factors and personal variables that account for intracultural variation of medicinal plant knowledge in the Andean highlands? and (2) what are the dominant modes of knowledge transmission? Our first working hypothesis was that schooling, migratory activity, and market integration—all important factors of social change in the Andes-influence the distribution of medicinal plant knowledge among rural Andean communities. Our second hypothesis was that medicinal plant knowledge is mainly transmitted vertically from parents or grandparents to offspring—a transmission mode typical of conservative knowledge systems with high intracultural variation [11].

## 2. Materials and Methods

Research for the present paper was part of BioAndes, a regional program of the Swiss Agency for Development and Cooperation that aimed to conserve biocultural diversity in Andean areas of Peru, Bolivia, and Ecuador. BioAndes was executed by a consortium of local institutions and their network of local partners from November, 2005 to March, 2011 and included conservation projects and action-research activities (for a synthesis of BioAndes experiences see [40]). Agreements were signed between BioAndes and the municipal governments of the geographic areas in which this program was implemented, prior to the realization of the activities.

### 2.1. Study Sites

**Figure 1:**
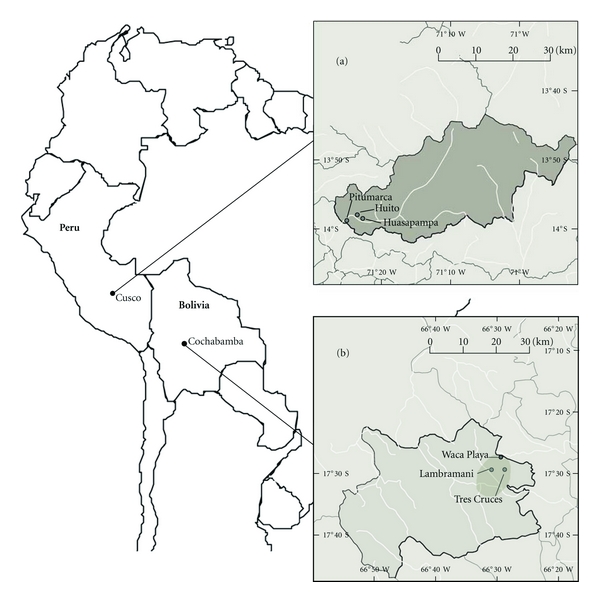
Map of the study sites. Research was conducted in two communities from Pitumarca District in the Department of Cusco, Peru, (a) and in two communities from Waca Playa Subcentral in the Province of Tapacari, Department of Cochabamba, Bolivia, (b).

For the purpose of this study, two of the seven implementation areas of BioAndes were selected as case study sites owing to their similarity in terms of ecological and cultural settings: the district of Pitumarca in the Department of Cusco in Peru and the subcentral of Waca Playa in the Department of Cochabamba in Bolivia (Figure 1). Both have comparable biogeographic characteristics, the main difference being the dryer climate and lower altitude of the Bolivian site. In both areas, the natural vegetation has been seriously affected by human activity. The vegetation is mainly composed of shrubs, grasses, remnant patches of native *Polylepis* spp. forests, and exotic plantations (such as *Eucalyptus* spp. and *Pinus* spp.). Both areas are inhabited by Quechua-speaking indigenous farmers primarily engaged in small-scale subsistence farming and are characterized by high rates of temporal and permanent migration to urban centers and Amazonian lowlands. Agricultural activity is quite comparable and varies according to altitudinal belts: growing of grains and cereals (maize, wheat, barley, oats, and quinoa), vegetable and fruits, potatoes and other Andean tubers such as *Ullucus tuberosus *Caldas and *Oxalis* spp., and livestock herding (sheep, cows, and goats in Waca Playa and lamas and alpacas in the highlands of Pitumarca). At both sites the population is socially organized into peasant communities that were created after the agrarian reforms that took place in both countries (from 1968 to 1975 in Peru and in 1953 in Bolivia), when most *haciendas* (large land holdings of Spanish and other immigrant descendants) were dismantled, and land was distributed among the indigenous farmers. Andean medicine is prevalent in both study areas, with the existence of specialists such as healers (called *yachayniuq* or *p'aqo* in Pitumarca and* yatiri* in Waca Paya), midwives, and bone setters. Lay people also possess important knowledge about traditional remedies and self-medicate mostly by using plants collected locally and also animals and minerals [45–47].

Pitumarca District is located in Canchis Province, 87 km south-west of the city of Cusco, in the Southern Peruvian highlands. Altitude ranges from 3,400 meters above sea level in the valley to 6,372 meters above sea level at the Ausangate summit. The climate ranges from semihumid, temperate cold, to humid and frigid, with mean annual temperatures and precipitation varying according to altitude, from 12°C and 650 mm in Pitumarca (3,600 meters above sea level) to 8°C and 910 mm in Phinaya (situated at 4,500 meters above sea level) [45]. Precipitation is concentrated during the rainy season from November to February. The area belongs to the “Central Andean Wet Puna” ecoregion, characterized by montane grassland and shrubland biome [48]. The district is composed of a total of eleven peasant communities, which, in turn, are divided into “annexes” and “sectors”. Research was carried out in two adjacent villages, which are, formally speaking, *anexos* (annexes) of peasant communities. The *anexo* Huasapampa (community of Pitumarca-Consachapi) is situated at 13°58′30′′S and 71°22′41′′W, at 3,700 meters above sea level, and is composed of 63 households. The *anexo* Huito (community of Pampachiri) is situated at 13°57′57′′S and 71°23′29′′W, at 3,680 meters above sea level, and is inhabited by 61 households. Both villages are located in the lower zone of Pitumarca's watershed, at 4 km from the district capital. At Pitumarca's weekly market on Saturdays, farmers sell their surplus production and merchants trade manufactured goods. Formal education is provided in the Spanish language in the *anexos* up to 6th grade and in Pitumarca up to the 12th grade. There is a health centre in the district capital with one medical doctor, two nurses, two obstetricians, and 6 auxiliary nurses. Each *anexo* is under the supervision of one auxiliary nurse, who visits the families once a month and trains community health workers in nutrition, hygiene, breastfeeding, and prevention of common afflictions such as parasitical illnesses, pneumonias, and diarrhea.

Waca Playa Subcentral belongs to Tunari National Park in Tapacari Province, 65 km east of the city of Cochabamba, in the Eastern cordillera of the Bolivian Andes. The altitude ranges from 2,760 to 4,100 meters above sea level. The climate is semiarid to semihumid and temperate cold, with a mean annual temperature of 11°C and mean average precipitation of approximately 500 mm that is distributed throughout the rainy season from November to March [47]. According to Olson et al. [48], the site corresponds to two global ecoregions and their corresponding biomes: the “Central Andean Puna” above 3,200 meters above sea level (montane grassland and shrubland) and the “Bolivian Montane Dry Forests” below 200 meters above sea level (tropical and subtropical dry broadleaf forests). Waca Playa is composed of five peasant communities, in two of which data were collected for the present study: the community of Tres Cruces (17°29′30′′S and 66°28′34′′W; 3,330 meters above sea level) inhabited by 49 households and the community of Lambramani (17°29′31′′S and 66°30′44′′W; 3,450 meters above sea level) with 40 households. The two communities are located 9 km (Tres Cruces) and 5 km (Lambramani) from Waca Playa respectively, where inhabitants from the communities and neighboring subcentrals meet at the weekly market to sell and exchange products. Schooling is offered in Spanish up to the 3rd and 6th grades in the communities (in Lambramani and Tres Cruces, resp.), and up to the 8th grade in Waca Playa. Since 1995, primary health care has been available at a health post in Waca Playa, where one auxiliary nurse operates. This health post provides limited healthcare to a total of 20 peasant communities from the three subcentrals of Waca Playa, Jatun Cienega, and Lapiani. Basic health care such as contraceptives, vaccination, painkillers, and antibiotics are provided. The auxiliary nurse also visits the communities, approximately once every two months. In case of serious illnesses or accidents people are sent to the hospital in the towns of Sipe Sipe, Vinto, or Quillacollo, approximately 40 to 50 km from Waca Playa.

### 2.2. Field Work and Selection of Participants

Field work was performed by the first author during numerous visits to the two case study sites between June, 2006 and April, 2010. Most data collection took place between March, 2007 and December, 2008. Logistic support was given by partners of BioAndes who already had a long working presence in these areas, namely, AGRUCO in Waca Playa and CEPROSI, IMAGEN, and IMAPI in Pitumarca. In each of the four communities, initial meetings were facilitated by these organizations in the local language to present the research aims and select the participating households. At these community meetings, it was also jointly decided that research results would be returned to the participants by means of the elaboration of medicinal plant booklets at each research site. Except in Huito, where there was no community consensus about authorizing the research because a few individuals expressed concern about the possible economic motivation of the study, community members from Huasapampa, Tres Cruces, and Lambramani all gave their collective verbal consent for carrying out the proposed research activities. In Huito, individual verbal consent was granted by the participants, who either voluntarily approached the first author to contribute to the research, or were approached by her and selected through snowball sampling.

The criteria for selection of participants were age and residence: each community assembly was asked to make a selection representing young (recently married and/or with small children), middle-aged (with grown-up children that participate in family tasks), and elderly households (couples or widows whose children had already left home). In Huito the same criteria were applied to selection of volunteers. Furthermore, in Waca Playa where the population is scattered over the territory, an additional criterion was to include an equal number of participants who lived in the communities' upper, middle, and lower zones.

### 2.3. Household Interviews

Semistructured interviews and freelisting exercises were conducted with 18 households from Pitumarca (9 households each from the Tres Cruces and Lambramani community, or 18% and 23% of all households, resp.) and 18 households from Waca Playa (10 households from Huasapampa and 8 households from the Huito community, or 16 and 13% of all households, resp.). The data were collected in three to six visits to each household, depending on the participants' availability, meaning that it was often impossible to complete the interview during one visit and that it continued during subsequent visits.

Interviews were conducted in Quechua, Spanish, or in both languages, according to the participants' preference and language ability, with the help of a native Quechua-speaking translator. The husband and/or the wife—and in some cases the children also—were asked questions about the household's characteristics, history, and livelihood strategies. In addition, the adult most knowledgeable about medicinal plants (husband or wife), according to the household members' own perceptions, was asked to list all the medicinal plants (s)he knew and/or used and explain how (s)he acquired his/her knowledge about natural remedies and Andean medicine in general. (S)he was also asked to include the medicinal plants that grew outside the research site, such as dry plants from the Amazon that they bought at the local markets or plants from the highlands that they exchanged with members of other communities. Interviews were conducted individually with the most knowledgeable member, but in about 50% of the cases, other members of the household unit (spouse and/or children) were present and allowed to intervene. This happened because interviews were usually conducted during the household's daily activities, during which other members were present; these activities included cooking and eating, washing clothes, resting during a day work in the fields, or grazing livestock. It was thus difficult to either systematically isolate only the most knowledgeable individual from the other household members, or to systematically ensure that all household members would be present.

Most interviews were recorded and translated into Spanish when they were conducted in Quechua and then transcribed. In the few cases when participants' authorization was not granted for recording or when it was technically not possible to do this, detailed notes were taken during the interviews in Spanish and eventually transcribed.

### 2.4. Plant Collection and Identification

Voucher specimens of most medicinal plants were collected and photographed at the two research sites during walks with participants. Information was recorded about the collection number, date, locality, informant, and the plants' local names. At the end of the day, specimens were pressed and dried according to standard botanical practices. In cases of contradictory information from different participants about a plant's name, additional specimens were collected in order to double-check the information. Cultivated and broadly distributed medicinal plants (such as *coca-Erythroxylum coca* Lam.) as well as rare or unavailable plants (plants at higher altitudes or in the Amazon lowlands, for instance) were not collected.

Voucher specimens were identified by specialists at the Herbarium Vargas from the *Universidad Nacional de San Antonio Abad del Cusco* in Cusco, Peru, and at the National Herbarium Martin Cardenas from the *Universidad Mayor de San Simón* in Cochabamba, Bolivia. When no voucher specimen was collected, the plants were indirectly identified on the basis of a comparison of their local names, biological and ecological characteristics, and medicinal uses with existing literature [44, 49, 50]. Plant scientific names and author names were verified for their correctness using the IPNI [51] and Tropicos.org [52] databases.

### 2.5. Data Analysis

Data were recorded at the household level, because preliminary field trips and observation showed that the household was the basic unit for health care practices at both research sites. When a household member is ill, the most knowledgeable parent usually diagnoses the ailment and decides on the treatment that will be administered—in some cases alone but frequently in consultation with the other parent. If this treatment includes the use of medicinal plants, which is frequently the case, then the children are often asked to collect these plants. As a consequence, knowledge about medicinal plants is widely shared and discussed at the household level, which also explains why the interviews were sometimes conducted with several household members.

The socioeconomic characteristics of participating households were compiled from interview transcriptions. The following variables were taken into account: community of residence, age, sex, kinship ties, level of education, migratory activity, degree of market integration, and health specialization. For the purpose of analysis, we distinguished the following three age categories according to the age of the main household member interviewed: (1) young households (20 to 34 years old), (2) middle-aged households (35 to 49 years old), and (3) old households (50 years old and above). We recorded the sex of the main household member interviewed. Where they existed, we recorded the first degree kinship ties between the main interviewee in each household (parents-child, grandparents-grandchild, aunt/uncle-niece/nephew, and between siblings, including in-laws). The level of education was measured as the level achieved by the main household member interviewed: (1) none (no formal education), (2) primary level (1 to 6 years of schooling), (3) secondary level (7 to 12 years of schooling), and (4) superior level (13 years of schooling and above). We distinguished three categories according to household migratory activity: (1) households with no migratory activity (who only made short trips to neighboring towns or communities to visit relatives, sell and buy products, or go to a health center), (2) seasonal migrants (who spend or in previous years had spent up to three months per year either in the lowlands as agricultural workers or merchants, or in neighboring urban centers to pursue off-farm activities); and (3) semipermanent migrants (who had lived for over one year outside the research area). We also classified participating households into three categories according to their market integration: (1) low (agricultural production mainly for own consumption, only surplus and, when necessary, one sheep or head of cattle sold at the local weekly market), (2) moderate (regular sale of agricultural products, sheep, own made wool, cheese or textiles on the local market and, in some cases, also at important commercial centers in the area), (3) and high (besides farm activities, these households traded cattle at important commercial centers in the area, made regular trading trips to the Amazon lowlands, and/or had a small store in the community). When households identified themselves and were identified by other community members as specialists (healers practicing Andean medicine or health workers collaborating with the health center) this information was also recorded.

On the basis of freelisting exercises, the number of medicinal plants mentioned by each household was recorded and taken as an indicator of plant knowledge. When several household members were interviewed, the plants that were cited by the various participants were summed. The number of medicinal plants mentioned thus corresponds to the household's total knowledge (at both study sites, the most knowledgeable household member contributed approximately 85% of this information as opposed to other household members). Furthermore, at each study site, an inventory of medicinal plants locally known and/or used by the 18 participating households was compiled. To identify the best-known medicinal plants, the number of households that mentioned each plant was tallied, and plants were ranked accordingly.

Comparison of the average or median medicinal plant knowledge among households was carried out in two ways. First, the amount of plant knowledge, calculated as the average or median number of plants mentioned per household, was compared at each research site according to the household's socioeconomic characteristics. The correlation between age and the total number of medicinal plants mentioned per household was statistically tested by means of the Spearman Rank Order Correlation in Pitumarca (because normality of data failed) and the Pearson Product Moment Correlation in Waca Playa (because the data were normally distributed). The categories of community of residence, sex of the main interviewee, level of education of main interviewee, migratory activity, and market integration were compared statistically by means of a *t*-test when data were normally distributed and equal variance testing passed, or alternatively with the Mann-Whitney Rank Sum test when one of these conditions was not met. Categories were only compared statistically when at least 5 households were involved, which was the case at both study sites for households according to their community of residence, households with seasonal versus semipermanent migration, and households with low versus moderate or high market integration. Households with no migratory activity (3 households in Pitumarca and 5 in Waca Playa) were thus pooled out of the analysis, and households with moderate and high market integration were merged into one category. Only in Pitumarca was it possible to compare households with primary education versus secondary education or higher, since in Waca Playa, there was only one household with secondary education or higher. At both sites, households' medicinal plant knowledge was not compared according to kinship ties or health specialization, because there were less than 5 households involved in each category.

Second, the type of medicinal plant knowledge was compared using cluster analysis (NTSYpc21 version 2.10 L) as used by [26], taking into account the plants mentioned by at least one third of all participants at each research site (6 of the 18 households from Pitumarca and 6 of the 18 households from Waca Playa). Cluster analysis is a useful tool for identifying pairs of participants with a high degree of agreement (on medicinal plants in our case) and kinship relations—which are, in turn, evidence of vertical and horizontal modes of cultural transmission [26]. Data were entered on an Excel spreadsheet with plant names as rows and households as columns, and cells contained the value “1” if the household mentioned the plant or “0” if it did not. The spreadsheet was imported into NTSYpc21 and the Dice coefficient was used to produce a matrix of (dis)similarity between pairs of households. A tree was then generated using the UPGMA-SAHN method. A correlation coefficient “*r*” (normalized Mantel statistic *Z*) was calculated to measure the correspondence between the tree matrix and the original data. The degree of fit of the cluster analysis was interpreted as follows: 0.9 ≤ *r*, very good fit; 0.8 ≤ *r* < 0.9, good fit; 0.7 ≤ *r* < 0.8, poor fit; *r* < 0.7, very poor fit. The clusters revealed by the trees were then interpreted in light of the household's socioeconomic characteristics, in order to identify patterns in the distribution of medicinal plant knowledge at each case study site.

The household interview transcriptions were analyzed qualitatively to assess the processes of knowledge transmission and acquisition. Answers about the source of acquisition of medicinal knowledge were summed according to the following categories, corresponding to the modes of cultural transmission described by Hewlett and Cavalli-Sforza [11]: (1) parents, grandparents (vertical transmission), (2) neighbors, extended family (horizontal transmission), (3) healers, specialists (horizontal transmission, “one-to-many” type), (4) elder (horizontal transmission, “many-to-one” type), and (5) supernatural origin, books, NGOs (nonpersonal modes of transmission). Likewise, the answers about the time of acquisition of plant knowledge were summed according to two categories: (1) childhood and (2) adulthood.

## 3. Results

### 3.1. Participating Households' Socioeconomic Characteristics and Medicinal Plant Knowledge

The socioeconomic characteristics and medicinal plant knowledge of the participating households are summarized in Tables 1 (data from Pitumarca) and 2 (data from Waca Playa). They include residence, age, age category, sex, kinship ties, education level, migratory activity, market integration, health specialization, and number of medicinal plants mentioned. The number of plants mentioned was elicited from the freelisting exercises. In both Pitumarca and Waca Playa, there were important variations in the amount of medicinal plant knowledge among participating households (range of 12–99 medicinal plants in Pitumarca, and 15–50 in Waca Playa).

No significant differences were found between the average or median number of medicinal plants mentioned by households according to the community of residence in either Pitumarca (Huasampa versus Huito communities; Mann-Whitney Rank Sum Test, *T* = 74.5) or Waca Playa (Tres Cruces versus Lambramani communities; *t*-test, *t* = 1.082). No correlation between age of the main interviewee and number of plants mentioned was found in Pitumarca (Spearman Rank Order Correlation) or in Waca Playa (Pearson Product Moment Correlation). Neither in Pitumarca nor in Waca Playa were there statistical differences between the median number of plants mentioned by households according to the sex of the main person interviewed (*t*-test; Pitumarca: *t* = 1.819, Waca Playa: *t* = 0.552). In Pitumarca there was no statistical difference (Mann-Whitney Rank Sum Test, *T* = 44.500) between the median number of plants mentioned by households that had only a primary education as compared to households that had a secondary education or higher. No differences in the amount of plant knowledge were found in either Pitumarca or Waca Playa when comparing households on the basis of seasonal versus semipermanent migration (Pitumarca: Mann-Whitney Rank Sum Test, *T* = 47.00; Waca Playa: *t*-test, *t* = 0.0414), or households with low market integration versus moderate or high integration (Pitumarca: *t*-test, *t* = −1.138; Waca Playa: *t*-test, *t* = 0.918). In summary, at both study sites, the amount of medicinal plant knowledge was not influenced by socioeconomic factors such as the community of residence, age, sex, level of education, migratory activity, or market integration.

**Figure 2:**
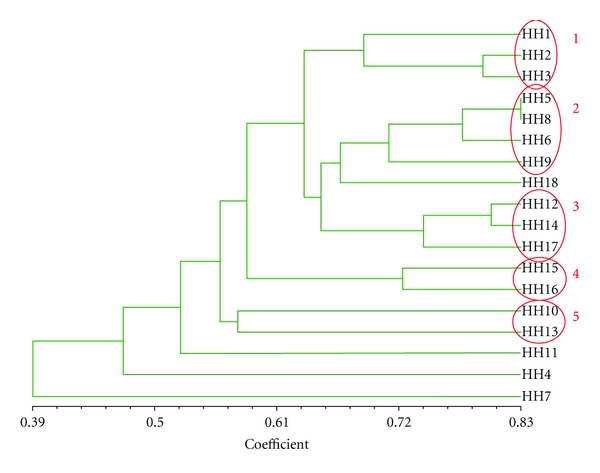
Tree resulting from cluster analysis of 18 households' knowledge about 47 medicinal plants in Pitumarca, Peru. HH no.: household number. Correlation coefficient *r* = 0.81 (good fit).

In Pitumarca, participants mentioned a total of 249 medicinal plants that they knew and/or used in the free listing exercises. The 47 best-known plants (those that were mentioned by at least 6 of the 18 participating households) were used to compare households' medicinal plant knowledge with cluster analysis and are listed in Table 3. The tree resulting from the analysis (Figure 2) reveals four main clusters of households based on the similarity of their knowledge about medicinal plants. These clusters, when triangulated with the data from the households' socioeconomic characteristics presented above, can be interpreted as follows.

1. Young households from the Huasapampa community, with seasonal or no migratory activity and low market integration; the cluster includes two siblings (in-laws).
2. Old households from the Huasapampa community, with seasonal migratory activity and low market integration (apart from household 6, which is middle-aged and showed a high level of market integration); the two traditional healers, who are also siblings, are part of this cluster.
3. Households from all age categories from the Huito community, with seasonal or no migratory activity and low market integration.
4. Two middle-aged households, both from the Huito community, whose main interviewee was male, with semipermanent migratory activity and moderate market integration.

A fifth cluster of two households was identified even though the association between them is weak.

(5) One middle-aged and one old household, whose main interviewee was male, both with semipermanent migratory activity and moderate to high market integration.

Households were not grouped according to the sex of the main person interviewed (with the exception of two households from cluster 4 and the two households from cluster 5), nor were households that had a parents-offspring or grandparents-grandchild kinship relationship. Moreover, the household's levels of education showed great heterogeneity in the described clusters.

In summary, the analysis revealed the following patterns in the data: the factors that influence the distribution of medicinal plant knowledge in Pitumarca are community of residence, age category, intragenerational kinship ties, migratory activity, market integration, and health specialization in the case of Andean healers. The factors that did not have an influence on medicinal plant knowledge distribution were sex, intergenerational kinship ties, level of education, and health specialization in the case of health workers.

**Figure 3:**
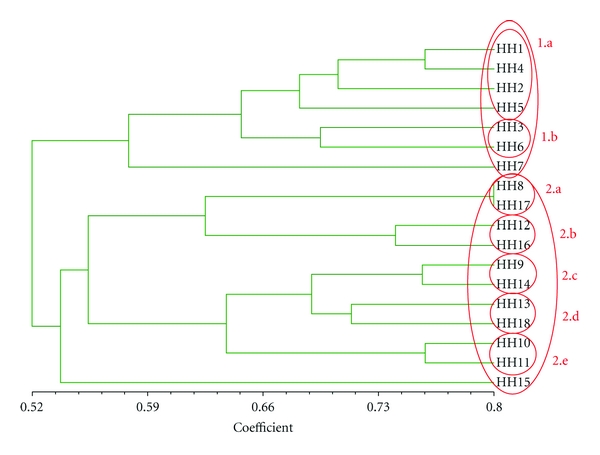
Tree resulting from cluster analysis of 18 households' knowledge about 25 medicinal plants in Waca Playa, Bolivia. HH no.: household number. Correlation coefficient *r* = 0.64 (poor fit).

The participating households from Waca Playa cited a total of 150 medicinal plants. The 25 best known plants of this list (those mentioned by at least 6 of the 18 households) were used to run the cluster analysis (see Table 4).  Figure 3 shows that the households from Waca Playa, on the basis of their knowledge about medicinal plants, were grouped into two main clusters, which in turn are divided into subclusters as follows:

1. Households from the Tres Cruces community.
  - Young and middle-aged households, with seasonal or no migratory activity and low to moderate market integration.
  - Two old households with seasonal or no migratory activity and low market integration.
2. Households from the Lambramani community (apart from households 8 and 9)
  - One middle-aged and one old household, whose main interviewee was male, both with semipermanent migratory activity and low to moderate market integration.
  - One middle-aged and one old household, with seasonal or no migratory activity, both with high market integration.
  - Two old households with seasonal or no migratory activity and low market integration; both are traditional healers.
  - Two old households, whose main interviewee was female, both with semipermanent migratory activity and moderate market integration.
  - Two middle-aged households with semipermanent migratory activity and low to moderate market integration.

According to this interpretation of the cluster analysis results, the factors that influence the distribution of medicinal plant knowledge in Waca Playa are the community of residence, age category, migratory activity, market integration, and health specialization. Sex, kinship ties (intra- and intergenerational) and education level do not influence medicinal plant knowledge distribution. Interestingly, these results confirm the patterns identified in Pitumarca.

### 3.2. Acquisition of Medicinal Plant Knowledge

**Figure 4:**
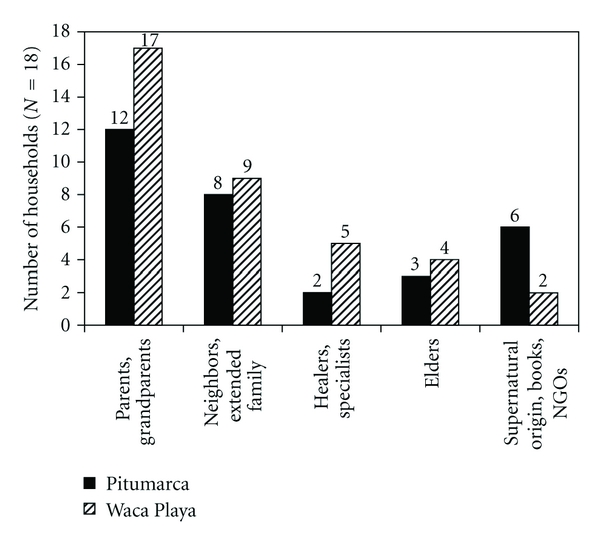
Sources of medicinal plant knowledge for 18 households from Pitumarca and 18 households from Waca Playa. Different sources were sometimes mentioned by one household, which explains why the sum is greater than 18 at each study site.

Figure 4 shows how participating households in the two case study areas acquired their medicinal plant knowledge. As the figure illustrates, households acquired their knowledge through a variety of sources. Vertical transmission (parents and grandparents) was mentioned by 67% and 94% of the households from Pitumarca and Waca Playa, respectively, (29 households in total), whereas horizontal transmission (neighbors, extended family, healers, specialists, and elders) was reported by 72% of the households from Pitumarca and by all the households from Waca Playa (31 households in total).

Six households from Pitumarca and five from Waca Playa learned about medicinal plants from a traditional healer or specialist (one-to-many mode of cultural transmission). But in the case of four of the households from Pitumarca, this specialist happened to be the parent or grandparent of the participant and was thus pooled out of the analysis, because it also accounts for a vertical mode of cultural transmission. Elders from the community were also reported as a source of knowledge (many-to-one transmission).

In addition to these interpersonal modes of cultural transmission, other types of knowledge sources were cited by the participants. The specialized knowledge of Andean healers, for instance, is usually believed to have a supernatural origin. Three (two from Pitumarca and one from Waca Playa) of the four specialists interviewed mentioned that their knowledge about traditional medicine could be explained by a *rayo* event (being struck by lightning), or by having been taught by the *apus* or *parajes* (sacred mountains that surround the communities). Four households (three from Pitumarca and one from Waca Playa) also mentioned that they became skilled in recent years through books or workshops about medicinal plants conducted by NGOs active in the area or through media such as local newspaper or radio broadcasts.

Regarding the time of acquisition of participants' medicinal plant knowledge, ten households from Pitumarca (56%) and fourteen from Waca Playa (78%) mentioned that they had learned as children, while twelve (67%) and eleven (61%) households from Pitumarca and Waca Playa, respectively, said that they acquired their skills as adults. Both times were sometimes mentioned by one household, which explains why the sum is greater than 18 at each study site. Here, also there seem to be equal portions of horizontally versus vertically acquired plant knowledge Knowledge about plant remedies is typically first acquired during childhood within the family (24 households in total), but an important part of this knowledge is also gained in adulthood, usually from people other than parents or grandparents (23 households).

As the quotations below demonstrate, there is a strong perception, widely shared among the participants, that medicinal plant knowledge is not “taught” as such, but it is the result of one's own personal active quest. In order to acquire medicinal knowledge, one needs to ask people in one's immediate environment, listen, look, try, and practice. And the capacity to learn will depend on interest, curiosity, intelligence, and in the end on personal ability.

*“Nobody taught me [about medicinal plants]; I learned on my own, looking at what other people were doing. (…) I do not teach my children; they learn only by looking. [I do not teach them] because they do not understand; but if they see they get to understand better. They always see.” (Waca Playa-Household 14, 29/04/2008)*

*“From the elders, looking at the elders I learned [about healing]. I got more interested and with practice learned more and more. Thanks to my intelligence and ability I got to learn (..) My children are intelligent; they will learn by looking at what I am doing.” (Pitumarca-Household 8, 29/07/2008)*

Medicinal plant knowledge is freely shared among community members out of solidarity and in a relationship of reciprocity, either through informal exchanges and comments during daily activities or when someone is ill and needs help from his/her neighbors or kin (see quotations below). In fact, the acquisition of medicinal plant knowledge is often the result of concrete illnesses that occur within the family, “as we walk through life”, and that oblige one to look for a specific remedy within the communal body of therapeutic knowledge. According to the households interviewed, this also explains why women are usually more knowledgeable about plant remedies than men, since they need to care for the children when they are ill.

“*When I went to the chacra [fields], I asked the elders [about medicinal plants] and they also asked me; this way we are sharing, this is our wisdom. (Pitumarca-Household 15, 01/10/2008)*”

*“I learned [about medicinal plants] since I got ill; the neighbors bring me the plants. (…) I do not teach them [my children]; on their own they learn when someone from the family gets ill. For example, since I got badly ill (…) my husband and my daughter learned to heal. (…) My daughter knows how to gather thurpa (Nototriches spp.), oq'e thurpa (Nototriches sp.), puka thurpa (Nototriches sp.), chili chili (Geranium filipes* Killip*), from faraway places up in the mountains; she knows much more about this than me. Because I was ill, she asked the people that live in the communities; so from various sources of information [she learned].” (Pitumarca-Household 5, 23/07/2008) *

The following narrative from a woman who grew up in town before coming back to the rural area where her parents lived illustrates that knowing how to use medicinal plants is considered to be among the skills needed to live in the community, skills which are not shared by people who live in urban centers.

“*When I was a child I did not know the medicinal plants. When I arrived here I saw my mother, she would heal my little sisters and my father when they would get ill. When I would get ill she also healed me, and by looking I learned.” (Waca Playa-Household 1, 01/12/2007) *

## 4. Discussion

### 4.1. Distribution of Medicinal Plant Knowledge

Our findings show that there is an important intracultural variation of medicinal plant knowledge in terms of the number of plants known at both study sites, but no clear patterns could be identified to explain this variation. Factors generally reported in the literature to account for differences in ethnobotanical knowledge and LK in general such as age [16, 21, 23, 53–56], sex [16, 18, 22, 23, 55, 57], schooling [9, 13, 15, 16, 18, 21, 30, 55], migration [42], or the market economy [34, 35, 41] did not influence the number of medicinal plants mentioned. A possible explanation for this result lies in important variations with respect to the level of specialization among participants. Indeed, the participants ranged from lay people with little knowledge to specialists. Arias Toledo et al. [17] encountered the same situation in the Cordoba region in Argentina. These findings imply that other factors, such as individual motivation, experience, and personality may play a more important role in influencing individual ethnobotanical knowledge than socioeconomic and other personal circumstances. This hypothesis is supported by our data which show that the acquisition of plant knowledge is the result of a personal quest and one's own interest and ability. These findings also suggest that Andean medicinal plant knowledge is not necessarily under threat of being lost due to factors of social change. This last hypothesis needs to be corroborated by complementary studies.

Contrary to our findings on the *amount* of plant knowledge, cluster analysis revealed clear patterns of variation in the *type* of medicinal plant knowledge at each study site and showed that the distribution of ethnobotanical knowledge was influenced by the community of residence, age, intragenerational kin relations, migratory activity, and market integration. Our study thus shows that knowledge about particular medicinal plants is highly patterned, even within one culturally homogeneous group living in the same biophysical territory. At each study site, households shared similar knowledge with their fellow community members, but demonstrated a type of knowledge distinct from households in neighboring communities. Other authors also describe Andean agricultural knowledge as “*art de la localité*” (“a local art”) and conclude that it is intimately associated with a particular place [7] and [58, page 209]. As a matter of fact, our study shows that medicinal plant knowledge is a skill needed to live in a particular community, as the example of a woman who had returned to the rural area after having lived in the city illustrated. Our interpretation of these results is that medicinal plant knowledge is a locally specialized resource that is part of the reciprocal exchanges that form the basis of Andean society [59] and that the extent to which this knowledge is shared within one community reflects the strength of its social organization. This was confirmed by the results showing the high importance of neighbors, elders, and the extended family in general in the transmission of knowledge about natural remedies, especially through their support during episodes of illness. Andean medicine is indeed one of the keystones of local society [60], and (community) specialization and reciprocity are among its structural features [59]. 

Another important finding from our study is that age peers, including siblings in the case of Pitumarca, have similar knowledge about medicinal plants, whereas this is not the case for kin from different generations, namely parents-children and grandparents-grandchildren. This result contradicts the hypothesis of a mainly vertical knowledge transmission. We suggest that life experience may be an important determinant of medicinal plant knowledge. People from the same generation are exposed to the same processes of change in the socioecological context throughout their lives (for instance, variable levels of migratory activity of the population, changes in the composition of vegetation or the status of natural resources, the presence of NGOs, etc.), and thus also to the same new sources of medicinal plant knowledge. (for instance, actors and contexts encountered during the migratory experience, new plants available locally, a workshop on medicinal plants conducted in the community, etc.). We believe that the prevalence of migration processes that affect all households at the study sites, but at different levels throughout the last decades, may account for the homogenization of plant knowledge among age peers. Another explanation is that age peers may face similar health problems and thus have the same need to know about natural remedies for treating them. Based on a survey conducted in Oaxaca, Mexico, Giovannini et al. [13] explain the positive correlation between age and knowledge and use of medicinal plants by the fact that people are more likely to become ill and be responsible for the health of others as they get older, and hence acquire more knowledge about medicinal plants. Our results also show that participants often acquired knowledge about specific plants when they faced a concrete illness, or were responsible for treating other household members, such as the case of women who care for young children.

The role of life experiences in individual medicinal plant knowledge can also explain our findings regarding the influence of migration and market integration. In Pitumarca and Waca Playa, households were often clustered according to their degree of migration and market activities. Both processes imply different degrees of mobility, since higher market integration meant traveling to the commercial centers of the region or to the Amazon lowlands. Consequently, both migration and market activities imply different degrees of interaction with noncommunity members, and thus access to new sources of medicinal plant knowledge. We postulate that the mobility of Andean households places them in situations of “encounters at multiple [social] interfaces” that stimulate the emergence of new knowledge as a product of the interaction between different actors [61]. Interestingly, our results show no difference in the amount of plant knowledge (number of plants reported) of households according to their migratory activity or degree of market integration. It thus remains to be elucidated whether the participating households have maintained their botanical knowledge or partially lost it and adopted new knowledge during their stay(s) outside the research sites. Nevertheless, the influence of mobility on medicinal plant knowledge is an indicator of the permeability of local medicinal systems by external influences. Ingold [62] describes knowledge about the world as a process of enskillment in the context of people's practical engagement with the environment. We believe that this is also true for migratory processes and that the attitude of a personal quest for medicinal plant knowledge described by the participants (by observing, questioning, and trying) is maintained and perhaps even enhanced during the migratory experience, giving continuity to the learning process in the host environment. In their study on medicinal plant use among Bolivian and Peruvian migrants in London, Ceuterick et al. [44] demonstrated how the resilience of Andean migrant communities includes processes of transformation, learning, reorganization, and renewal.

The fact that Andean healers at the study site share similar medicinal plant knowledge with their professional peers could be expected, since the distinction between specialist and lay knowledge is widely recognized in ethnomedicinal research around the world. In their paper on healers' knowledge in Bolivia, Vandebroek et al. [60] revised the literature about the specific modes of acquisition and transmission of specialist knowledge in several African and Latin-American countries. The three main sources of specialist knowledge include the family sphere, other experienced healers outside the family, and a supernatural origin (dreaming or communication with spirits). This was also observed in our study, where Andean healers reported that the source of their specialist knowledge was supernatural. We wish to draw attention to two additional aspects in our results. First, healers are not necessarily the most knowledgeable participants in their communities in terms of the total number of plants reported. At both study sites, some lay people actually possess a higher level of plant knowledge than the Andean specialists. Second, the clusters that include the healers in Pitumarca also include lay persons, and at both study sites, the clusters that contain halers are not isolated from the other clusters. These observations indicate that what differentiates Andean healers from lay people is not necessarily their high level of or exceptional idiosyncratic type of medicinal plant knowledge, but other personal attributes such as the supernatural origin of their knowledge.

Level of education had no influence on the distribution of the amount or type of medicinal plant knowledge among participating households, which contradicts previous findings about the negative correlation between schooling and LK [9, 13, 15, 16, 18, 21, 27, 30]. In line with Robinson [19], we believe that individual motivation has more influence on ethnobtanical knowledge than state-run education or other socioeconomic indicators of modernization. In the Argentinean Cordoba mountains, Arias Toledo et al. [17] reported on a group of persons with superior education, usually migrants from urban centers, who knew about a greater number of medicinal plants than their peers with less education. The former acquired their knowledge about plants through specialized books and training, or by asking local specialists. Similarly, participating households with the highest levels of education at both our research sites (secondary level or higher) exhibited a high personal interest in medicinal plants and expressed the desire to increase their knowledge by observing a healers, reading specialized books, or participating in workshops. Our findings imply that formal education does not compete with local forms of knowledge at our research sites. Not only do both systems coexist, but schooling may even indirectly strengthen medicinal plant knowledge.

### 4.2. Transmission of Medicinal Plant Knowledge

Our findings about the sources of acquisition of medicinal plant knowledge indicate that there are equal proportions of vertical versus horizontal modes of transmission at both study sites, which is a rejection of our working hypothesis and contradicts the results from other studies that state that LK is mainly transmitted by parents to offspring [3, 5, 11]. McElreath and Strimling [63] suggest that some ethnographic studies may have overestimated the importance of vertical transmission by focusing mainly on learning in children [63] (cited by [12]). Our own results support their position, since a comparable number of participants recalled that their teachers had been their parents or grandparents during childhood (vertical acquisition) as compared to those who had learned from other sources of knowledge during adulthood (horizontal acquisition). This may indicate that medicinal plant knowledge is first acquired within the family circle during childhood but that it then undergoes transformations as a result of subsequent contacts with other knowledge sources, including age peers. As Barsh argues, “every individual is necessarily engaged in a lifelong personal search for ecological understanding” [64, page 74]. Similar results about the increase of the importance of nonvertical transmission with age have also been reported in the literature [11, 12, 63].

Local healers represent another source of medicinal plant knowledge at our study sites (reported by almost one third of all participating households). Furthermore, the results from the cluster analysis indicate that Andean healers have a certain amount of medicinal plant knowledge in common with lay people from their communities. This observation suggests that healers are important actors in the cultural transmission of generalist knowledge, which represents a one-to-many mode of cultural transmission and thus of a highly dynamic knowledge system [11]. Lozada et al. [5] reported that experienced Mapuche healers used to play an important role in the transmission and conservation of plant knowledge in Northwestern Patagonia but that this was no longer the case and that knowledge transmission now occurred mainly vertically within the family. We might thus predict that the possible disappearance of these specialized healers, all elder people, in the next decades, could lead to transformations in the patterns of medicinal plant transmission. If the role of healers as knowledge transmitters is taken over by the older generation (either the parents or the community elders), then the change will be in the direction of a greater proportion of conservative transmission modes. Conversely, if the role of specialist is taken over by highly motivated and skilled individuals (that can be peers or neighbors from the young generation), this transformation would lead to a prevalence of dynamic transmission modes. As a matter of fact, our results also showed that other nonpersonal sources, linked to individual motivation and experience played a role in the acquisition of plant knowledge, and that these individual characteristics might indeed be the dominant factor of the amount of ethnobotanical knowledge.

### 4.3. Methodological Limitations of the Study

The methodological tool chosen for this research was freelisting. The advantage of freelisting and other types of open-ended questions frequently used in the social sciences is that responses from participants are not directed in any way by the researcher, thus allowing in our case for an unbiased inventory of the most significant plants known to and/or used by each participant. Freelists are useful for assessing who in a community knows more (or less) about medicinal plants [65]. The disadvantage of this tool is that a plant might not be mentioned, because it was simply “forgotten” during the exercise, as opposed to lack of knowledge about it. This can limit the subsequent use of statistical cluster analysis for comparison of knowledge between participants. The alternative would have been to undertake systematic data collection by means of a specimen identification task with each household, based on a selected list of medicinal plant vouchers (what Medeiros et al. [66] call a “check-list interview”). We have taken three measures to minimize methodological bias as a result of freelisting. First, interviews were carried out during several visits to each household, and in some cases, several household members were interviewed in order to obtain a complete list of known medicinal plants. Second, we used only the most frequently reported plants, assuming that these would be more widely known at the research sites. In fact, according to Martin [67], people tend to list the most culturally important plants first when asked to freelist. A limitation of this measure is that it disregards the lesser known plants, which can be interesting indicators of intracultural variation and knowledge transmission. However, these and other plants were taken into account for the comparison of the amounts of ethnobotanical knowledge (number of plants reported). Third, for the cluster analysis, we used the Dice coefficient to produce the (dis)similarity matrix between pairs of households. This coefficient puts a comparatively strong emphasis on the number of presence matches (number of plants known to each pair of households). This is not the case with other coefficients, such as, for instance, the simple matching coefficient that was used by other researchers to make similar cluster analyses [14].

A second methodological constraint of the present study was the limited sample size. A considerable amount of time was required to obtain qualitative data that provided in-depth overall knowledge about the research context and made it possible to establish relationships of trust with participants and conduct long open-ended interviews and informal exchanges. As a consequence, we collaborated with a total of 36 households at the two study sites over a total of 22 months. The same number of households was interviewed at each study site (18), in order to allow for comparison of the medicinal plant inventories from both sites. A larger sample size would be recommendable for a stronger statistical comparison of plant knowledge according to a set of differing socioeconomic variables.

Another aspect worth noting is the poor fit of the cluster analysis results from the Waca Playa case study. The correlation coefficient from the cluster analysis tree from Waca Playa was low (*r* = 0.64; poor fit), which means that the degree of correspondence of the analysis with the original data was weak, whereas the one from the Pitumarca tree was high (*r* = 0.81; good fit). Despite this, we included the results from Waca Playa in the present paper in order to verify whether the trends identified in the data from Pitumarca were confirmed. The data used in Waca Playa might be considered too limited to distinguish groups of clusters. The same analysis was thus run with a higher number of plants using the data sets from the two research sites (those mentioned by 17 and those mentioned by all 18 participating households), but the resulting correlation coefficients were even lower. One possible explanation for the weak results in Waca Playa could be the low agreement on medicinal plants among households, not allowing for strong patterns to be discovered. Defining culture as consensus, in line with Romney et al. [68], could offer an explanation for these results. According to these authors, participants who agree more with others are more knowledgeable. This was confirmed by our results, when comparing overall knowledge from Pitumarca with that in Waca Playa. In Pitumarca, where there was more agreement among households about commonly known medicinal plants, the total amount of medicinal plant knowledge was also greater (total of 249 plants known versus 150 in Waca Playa). A positive correlation between consensus and medicinal plant knowledge at the group level was also demonstrated by Vandebroek [26] although this correlation was negative at the individual level, namely, in the case of specialists who showed a low level of agreement with their peers but a high level of idiosyncratic knowledge.

A final constraint of this study is that voucher specimens of some plants could not be collected because of the study's time limitations, either because they grew far from the research site and were not easily available during the field work season, or because the specimens collected were deteriorated due to adverse climatic conditions. In these cases, the corresponding plants could only be determined indirectly through literature, which does not ensure the accuracy of this information and thus limits its use for future comparative studies with the same plants.

## 5. Conclusions

This study shows that LK in culturally homogeneous groups presents certain patterns of variation and distribution that result from differences at the socioeconomic and personal levels, including individual life experiences. Our findings demonstrate the usefulness of triangulating the results from several methods to assess intracultural variation, namely, by comparison of both the *amount* and *type *of ethnobotanical knowledge. On the one hand, comparison of the average or median number of plants mentioned per households did not allow us to identify clear patterns in the distribution of medicinal knowledge, but it provided clues about its resilience by demonstrating that factors of social change such as schooling, migration, or the market economy did not influence it. On the other hand, cluster analysis permitted a demonstration of the socioeconomic factors that explain more detailed differences and similarities between the types of knowledge in different households. In addition, assessment of the modes of transmission of medicinal plant knowledge was a valuable tool for better understanding of the dynamics of Andean LK. Future studies should investigate the transformations of the patterns of Andean environmental knowledge distribution and transmission over time. Processes of social change might indeed lead to a redefinition of the weight of the different factors that influence LK and modes of knowledge transmission. This kind of approach could provide valuable insights into the degree of resilience of Andean LK in a rapidly changing socioecological context.

## Figures and Tables

**Table 1:** Socioeconomic characteristics and medicinal plant knowledge of participating households from Pitumarca, Peru. Medicinal plants: total number of medicinal plants mentioned.

	Residence	Age (years)	Age category	Sex	First degree kinship ties	Education level	Migratory activity	Market integration	Health specialization	Medicinal plants
Household 1	Huasapampa	26	young	male	grandson of 8 and brother-in-law of 2	primary	seasonal	low	none	37
Household 2	Huasapampa	25	young	female	sister-in-law of 1	primary	seasonal	low	none	36
Household 3	Huasapampa	21	young	female	daughter of 5	superior	none	low	none	57
Household 4	Huasapampa	49	middle	male		primary	seasonal	high	none	22
Household 5	Huasapampa	52	middle	female	mother of 3	primary	seasonal	moderate	none	99
Household 6	Huasapampa	45	middle	female		primary	seasonal	high	none	50
Household 7	Huasapampa	44	middle	male		primary	seasonal	low	health worker	12
Household 8	Huasapampa	63	old	male	grandfather of 1 and brother of 9	primary	seasonal	low	Andean healer	82
Household 9	Huasapampa	76	old	male	brother of 8	primary	seasonal	low	Andean healer	44
Household 10	Huasapampa	62	old	male		primary	semipermanent	moderate	health worker	52
Household 11	Huito	32	young	male		secondary	semipermanent	low	none	28
Household 12	Huito	24	young	female		secondary	none	low	none	47
Household 13	Huito	38	middle	male		secondary	semipermanent	high	none	53
Household 14	Huito	43	middle	female		primary	seasonal	low	none	50
Household 15	Huito	42	young	male		primary	semipermanent	moderate	health worker	53
Household 16	Huito	47	middle	male		secondary	semipermanent	moderate	none	46
Household 17	Huito	68	old	male		none	seasonal	low	none	29
Household 18	Huito	65	old	female		none	none	low	none	47

**Table 2:** Socioeconomic characteristics and medicinal plant knowledge of participating households from Waca Playa, Bolivia. Medicinal plants: total number of medicinal plants mentioned.

	Residence	Age (years)	Age category	Sex	First degree kinship ties	Education level	Migratory activity	Market integration	Health specialization	Medicinal plants
Household 1	Tres Cruces	27	young	female	daughter of 9	primary	seasonal	low	none	27
Household 2	Tres Cruces	38	middle	female		primary	none	moderate	none	21
Household 3	Tres Cruces	51	old	male		primary	seasonal	low	none	23
Household 4	Tres Cruces	29	young	male		primary	seasonal	moderate	none	39
Household 5	Tres Cruces	39	middle	male	brother-in-law of 7, nephew of 6	primary	seasonal	moderate	none	17
Household 6	Tres Cruces	59	old	female	sister-in-law of 8, aunt of 5	primary	none	low	none	25
Household 7	Tres Cruces	38	middle	male	brother-in-law of 5	secondary	seasonal	low	none	15
Household 8	Tres Cruces	52	old	male	brother-in-law of 6	primary	semipermanent	moderate	none	16
Household 9	Tres Cruces	71	old	male	father of 1	primary	none	low	Andean healer	42
Household 10	Lambramani	43	middle	male		primary	semipermanent	low	none	27
Household 11	Lambramani	38	middle	female		none	semipermanent	moderate	none	32
Household 12	Lambramani	57	old	female		none	seasonal	high	none	24
Household 13	Lambramani	50	old	female		none	semipermanent	moderate	none	26
Household 14	Lambramani	50	old	female	niece of 15	none	seasonal	low	Andean healer	50
Household 15	Lambramani	63	old	female	aunt of 14	none	none	low	none	22
Household 16	Lambramani	38	middle	male		primary	none	high	none	23
Household 17	Lambramani	44	middle	male	brother-in-law of 18	primary	semipermanent	low	none	33
Household 18	Lambramani	54	old	female	sister-in-law of 17	none	semipermanent	moderate	none	47

**Table 3:** Medicinal plants from Pitumarca mentioned by 6 or more households (*N* = 18). Households: number of households who mentioned the plant. Voucher numbers: SM no. (P) = Sarah-Lan Mathez-Stiefel no. (Peru). Source: source of indirect scientific name identification: [a]  =  photograph taken by SM, [b]  =  [44], [c]  =  [50].

Local name(s)	Scientific name	Plant family	Households	Voucher numbers [or source]
ajinco	*Artemisia absinthium* L.	Asteraceae	7	SM73(P)
alqo kiska	*Acanthoxanthium ambrosioides (*Hook. and Arn.) D. Loeve	Asteraceae	10	SM10(P)
cáncer qhora	*Stachys* spp. (*S. arvensis* L., *S. herrerae* Epling)	Lamiaceae	8	SM123(P)-SM48(P)
cebada	*Hordeum vulgare* L.	Poaceae	6	SM122(P)
chiqchi	*Berberis carinata* Lechl.	Berberidaceae	6	SM35(P)
chirichiri	*Grindelia boliviana* Rusby	Asteraceae	15	SM91(P)
clavel, clavel rojo, clavel negro	*Dianthus* sp.	Caryophyllaceae	7	SM83(P)
coca	*Erythroxylum coca* Lam.	Erythroxylaceae	15	[b]
eucalipto	*Eucalyptus globulus* Labill.	Myrtaceae	16	SM78(P)
hierbabuena	*Mentha viridis *(L.) L.	Lamiaceae	9	SM5(P)
hinojo	*Foeniculum vulgare* Mill.	Apiaceae	6	SM51(P)
kanlli	*Margyricarpus pinnatus* (Lam.) Kuntze	Rosaceae	8	SM89(P)
kharo	*Colletia spinosissima* J. F. Gmel.	Rhamnaceae	12	SM21(P)
limón	*Citrus aurantiifolia *(Christm.) Swingle	Rutaceae	6	[b]
llantén	*Plantago major* L.	Plantaginaceae	6	SM96(P)
llauli, china llauli	*Barnadesia horrida* Muschl.	Asteraceae	14	SM26(P)
malva	*Malvastrum* sp.	Malvaceae	11	SM18(P)
manka phaki	*Ageratina sternbergiana* (D.C.) R. M. King and H. Rob.	Asteraceae	10	SM27(P)-SM109 (P)
manzanilla	*Matricaria recutita* L.	Asteraceae	15	SM82(P)
matapalo	*Gaiadendron *spp. *(G. punctatum* G. Don*, G. Ellipticum *(Ruiz and Pav.) Baehni ex. J. F. Macbr.)	Loranthaceae	6	[c]
muña	*Minthostachys* spp. (*M. setosa* (Briq.) Epling, *M. spicata* (Benth.) Epling	Lamiaceae	13	SM33(P)-SM120(P)
muñak'a	*Muehlenbeckia volcanica* (Benth.) Endl.	Polygonaceae	8	SM31(P)
mutuy	*Senna birostris *(Vogel) H.S. Irwin and Barneby	Caesalpinaceae	13	SM20(P)
nabo, yuyo	*Brassica rapa* subsp. *campestris* (L.) A. R. Clapham	Brassicaceae	11	SM4(P)
oq'e thurpa	*Nototriches *sp.	Malvaceae	6	[c]
oqororo, alqo oqororo	*Mimulus glabratus* Kunth	Scrophulariaceae	10	SM19(P), SM38(P)
orqo llauli	*Dasyphyllum leiocephalum* (Wedd.) Cabrera	Asteraceae	7	SM77(P)
patakiska	*Austrocylindropuntia subulata* (Muehlenpf.) Backeb. subsp. *exaltata* (A. Berger) D. R. Hunt	Cactaceae	12	[a]
perejil	*Petroselinum sativum* Hoffm.	Apiaceae	9	SM9(P)
pilipili, diente de leõn	*Taraxacum officinale* F. H. Wigg.	Asteraceae	11	SM6(P)
pimpinilla	*Pimpinella anisum* L.	Rosaceae	7	SM79(P)
puka thurpa	*Nototriche *sp.	Malvaceae	8	[c]
puka t'ikaq kisa	*Cajophora cirsiifolia* C. Presl	Loasaceae	10	SM25(P)
p'uku p'uku	*Dichondra* sp.	Convolvulaceae	7	SM39(P)
pupusa	*Xenophyllum poposum* (Phil.) V. A. Funk	Asteraceae	9	SM93(P)
salvia	*Lepechinia meyenii* (Walp.) Epling	Lamiaceae	8	SM88(P), SM106(P)
sangre de grado	*Croton lechleri *Muell. Arg.	Euphorbiaceae	7	[b]
sasawi	*Leucheria daucifolia* (D. Don) Crisci	Asteraceae	16	SM94(P)
thurpa	*Nototriche *spp. (*N.matthewsii* A. W. Hill, *N. turritella* A. W. Hill, *N. herrerae* Ulbr. ex A. W. Hill, *N. Flabellata* (Wedd.) A. W. Hill)	Malvaceae	9	[c]
toronjil	*Melissa officinalis *L.	Lamiaceae	8	[b,c]
uña de gato	Undet.		6	
wamanlipa	*Senecio tephrosioides* Turcz.	Asteraceae	16	SM95(P)
waraqo	*Opuntia floccosa *Salm-Dyck	Cactaceae	10	[a]
wichullu	Undet.		14	
yana kisa	*Urtica urens* L.	Urticaceae	6	SM50(P)
yawarch'unka	*Oenothera multicaulis* Ruiz and Pav.	Onagraceae	17	SM63(P)
zaptilla, pucucho pucucho	*Calceolaria* spp. (*C. sparsiflora* Kunze, *C. virgata* Ruiz and Pav., *C. aurea* Pennell)	Calceolariaceae	9	SM1(P)-SM111(P)-SM110(P)

**Table 4:** Medicinal plants from Waca Playa, Bolivia, mentioned by 6 or more households (*N* = 18). Households: number of households who mentioned the plant. Voucher numbers: SM no. (B) = Sarah-Lan Mathez-Stiefel no. (Bolivia), RB no. = Regine Brandt no. Source: source of indirect scientific name identification: [a]  =  [44], [b]= [49].

Local name(s)	Scientific name	Plant family	Households	Voucher numbers [or source]
andres huaylla	*Cestrum parqui *L'Hér	Solanaceae	17	SM9(B), SM132(B), SM88(B)
chini muña, muña	*Clinopodium bolivianum* (Benth.) Kuntze	Lamiaceae	11	SM15(B), SM93(B)
coca	*Erythroxylum coca* Lam.	Erythroxylaceae	16	[a]
durazno	*Prunus persica* (L.) Batsch	Rosaceae	7	SM55(B)
kalisto, eucalipto	*Eucalyptus globulus* Labill.	Myrtaceae	16	SM6(B), SM14(B), SM87(B)
khara malva, malva	*Malva parviflora* L.	Malvaceae	8	SM32(B), SM33(B), SM73(B), SM83(B)
khara sapi, kharasa, leche leche	*Sonchus oleraceus *L.	Asteraceae	8	SM58(B), SM66(B), SM134(B)
k'oa muña, haya muña, muña	*Minthostachys ovata* (Briq.) Epling	Lamiaceae	11	SM10(B), SM76(B), SM57(B)
lanti lanti	*Plantago *spp. (*P. orbignyana *Steinh. ex Decne.,* P. lanceolata *L.)	Plantaginaceae	6	SM61(B), SM103(B)-SM125(B), SM129(B)
llave	*Tripodanthus acutifolius* (Ruiz and Pav.) Tiegh	Asteraceae	9	[b]
manzanilla	*Matricaria chamomilla *L.	Asteraceae	10	SM62(B)
molle	*Schinus molle* L.	Anacardiaceae	15	SM16(B), SM89(B)
paiqo	*Chenopodium ambrosioides* L.	Amaranthaceae	7	SM29(B), SM60(B), SM67(B), SM91(B)
raqacho, raqa raqa	*Lepechinia graveolens* (Regel) Epling	Lamiaceae	9	SM18(B)
romansa, lanti lanti	*Rumex *sp.	Polygonaceae	10	SM22(B), SM70(B)
salvia	Undet.		8	—
sauco	*Sambucus nigra* L. subsp. *peruviana* (Kunth) Bolli	Adoxaceae	7	SM75(B)
sira ch'ilka	Undet.		6	—
sira paiqo, ch'ini paiqo	*Chenopodium ambrosioides* L.	Amaranthaceae	14	SM128(B)
t'ola	*Baccharis dracunculifolia *DC.	Asteraceae	7	SM13(B), SM100(B)
uri uri	*Pluchea fastigiata* Griseb.	Asteraceae	7	RB47.14
verbena	*Verbena hispida* Ruiz and Pav.	Verbenaceae	8	SM72(B)
wacanwayo	*Iresine *aff. *diffusa *Humb. and Bonpl. ex Willd.	Amaranthaceae	8	SM113(B)
wira wira	*Gnaphalium dombeyanum* DC.	Asteraceae	8	SM94(B), SM127(B)
zapatilla	*Calceolaria engleriana* Kraenzl.	Calceolariaceae	6	SM21(B), SM78(B)
